# Supplementary material for: An image analysis pipeline to quantify the spatial distribution of cell markers in stroma-rich tumors
Source: Front Bioinform. 2025 Sep 5;5:1619790. doi: 10.3389/fbinf.2025.1619790 (PMC12446346; doi:10.3389/fbinf.2025.1619790)
Supplement: Supplementary file 1 [file DataSheet1.docx]

**Supplementary for:**

**An image analysis pipeline to quantify the spatial distribution of cell markers in stroma-rich tumors**

Antoine A. Ruzette^1^, Nina Kozlova^2,3^, Kayla A Cruz^2,4^, Taru Muranen^2,3^, and Simon F. Nørrelykke^1*^

**Affiliations:**

1. Department of Systems Biology, Harvard Medical School, Boston, Massachusetts, USA
2. Department of Medicine, Cancer Research Institute, Beth Israel Deaconess Medical Center, Boston, Massachusetts, USA
3. Harvard Medical School, Boston, Massachusetts, USA
4. Biological and Biomedical Sciences PhD Program, Harvard University, Boston, Massachusetts, USA

* Correspondence: [simon@hms.harvard.edu](mailto:simon@hms.harvard.edu)

**Github:** <https://github.com/HMS-IAC/stroma-spatial-analysis-web>

# **Supplementary tables and figures**

Numbers in tables were rounded up to the sixth significant decimal.

***AsPC NDRG1***

**Table S1:** Parameters of the fitted lognormal distribution for the ***FITC KER: Cytoplasm: Median*** intensity across all detected cells. The intensity threshold used for cell classification in each image is determined by translating the reference threshold percentile (marked with an *).

| **Image** | **Location** | **Shape** | **Scale** | **Threshold** |
| --- | --- | --- | --- | --- |
| **#1** | –707.011083 | 0.619916 | 3138.058254 | 300* |
| **#2** | –723.643241 | 0.603754 | 2949.356883 | 251.28 |
| **#3** | –793.205413 | 0.618143 | 3229.167415 | 246.42 |
| **#4** | –308.258962 | 0.681428 | 1840.263228 | 219.30 |
| **#5** | –1129.335995 | 0.558484 | 4068.679791 | 331.98 |

**Table S2:** Parameters of the fitted lognormal distribution for the ***CY5 pNDRG1: Cell: Max*** intensity across all detected cells. The intensity threshold used for cell classification in each image is determined by translating the reference threshold percentile (marked with an *)

| **Image** | **Location** | **Shape** | **Scale** | **Threshold** |
| --- | --- | --- | --- | --- |
| **#1** | 734.582782 | 1.361657 | 1067.563348 | 1200* |
| **#2** | 700.593146 | 1.238082 | 756.449602 | 1056.18 |
| **#3** | 703.018174 | 1.229991 | 828.940911 | 1094.61 |
| **#4** | 687.089880 | 1.151794 | 608.544201 | 988.61 |
| **#5** | 721.599776 | 1.516394 | 1077.707109 | 1149.14 |

**Table S3:** Parameters of the fitted lognormal distribution for the ***TRITC FN: Cell: Median*** intensity across all detected cells. The intensity threshold used for cell classification in each image is determined by translating the reference threshold percentile (marked with an *)

| **Image** | **Location** | **Shape** | **Scale** | **Threshold** |
| --- | --- | --- | --- | --- |
| **#1** | 1127.574751 | 0.680330 | 1557.668026 | 5000* |
| **#2** | 1073.706558 | 0.741478 | 1482.000040 | 5072.28 |
| **#3** | 913.571101 | 0.573779 | 1331.199930 | 3783.08 |
| **#4** | 884.531585 | 0.670759 | 876.069113 | 3034.75 |
| **#5** | 1276.548976 | 0.727041 | 1644.706573 | 5629.18 |

**Table S4:** Agreement percentages of single-threshold classifiers when compared to machine learning classification results for pNDRG1 images. The same thresholds from one image were applied to classify cells in other images. σ is the sample standard deviation. All values are in percentage (%).

| **ML classifier** | **Classifier using thresholds from image number:** | | | | |
| --- | --- | --- | --- | --- | --- |
| 87.7 | **#1** | **#2** | **#3** | **#4** | **#5** |
|  | 96.1 | 86.4 | 90.1 | 78.7 | 94.0 |
|  | µ = 89.06  σ = 6.88 | | | | |

***AsPC Ki67***

**Table S5:** Parameters of the fitted lognormal distribution for the ***FITC KER: Cytoplasm: Median*** intensity across all detected cells. The intensity threshold used for cell classification in each image is determined by translating the reference threshold percentile (marked with an *)

| **Image** | **Location** | **Shape** | **Scale** | **Threshold** |
| --- | --- | --- | --- | --- |
| **#1** | 288.717525 | 0.599332 | 1632.425859 | 650* |
| **#2** | -550.184474 | 0.564812 | 4726.485901 | 590.79 |
| **#3** | -9.668936 | 0.489416 | 2167.226010 | 622.80 |
| **#4** | -310.003757 | 0.416821 | 2126.460683 | 434.95 |
| **#5** | -340.941994 | 0.797041 | 8712.196935 | 831.45 |

**Table S6:** Parameters of the fitted lognormal distribution for the ***CY5 Ki67: Nucleus: Max*** intensity across all detected cells. The intensity threshold used for cell classification in each image is determined by translating the reference threshold percentile (marked with an *).

| **Image** | **Location** | **Shape** | **Scale** | **Threshold** |
| --- | --- | --- | --- | --- |
| **#1** | 286.724866 | 1.801891 | 469.496465 | 950* |
| **#2** | 282.490001 | 1.643360 | 474.312730 | 932.51 |
| **#3** | 348.348119 | 1.593198 | 752.831402 | 1370.18 |
| **#4** | 241.491744 | 1.597186 | 255.996654 | 589.23 |
| **#5** | 260.713033 | 1.705415 | 707.838623 | 1242.37 |

**Table S7:** Parameters of the fitted lognormal distribution for the ***FITC FN: Cell: Median*** intensity across all detected cells. The intensity threshold used for cell classification in each image is determined by translating the reference threshold percentile (marked with an *).

| **Image** | **Location** | **Shape** | **Scale** | **Threshold** |
| --- | --- | --- | --- | --- |
| **#1** | 695.439669 | 0.477924 | 1499.172311 | 4000* |
| **#2** | 1053.805650 | 0.663097 | 1866.380304 | 6641.85 |
| **#3** | 1258.215495 | 0.603436 | 1219.408835 | 4566.16 |
| **#4** | 564.392175 | 0.634543 | 718.958016 | 2617.70 |
| **#5** | 805.830558 | 0.649840 | 1221.342242 | 4383.30 |

**Table S8:** Agreement percentages of single-threshold classifiers when compared to machine learning classification results for Ki67 images. The same thresholds from one image were applied to classify cells in other images. σ is the sample standard deviation. All values are in percentage (%).

| **ML classifier** | **Classifier using thresholds from image number:** | | | | |
| --- | --- | --- | --- | --- | --- |
| 86.3 | **#1** | **#2** | **#3** | **#4** | **#5** |
|  | 84.2 | 83.7 | 92.3 | 66.6 | 90.4 |
|  | µ = 83.4  σ = 10.1 | | | | |

**
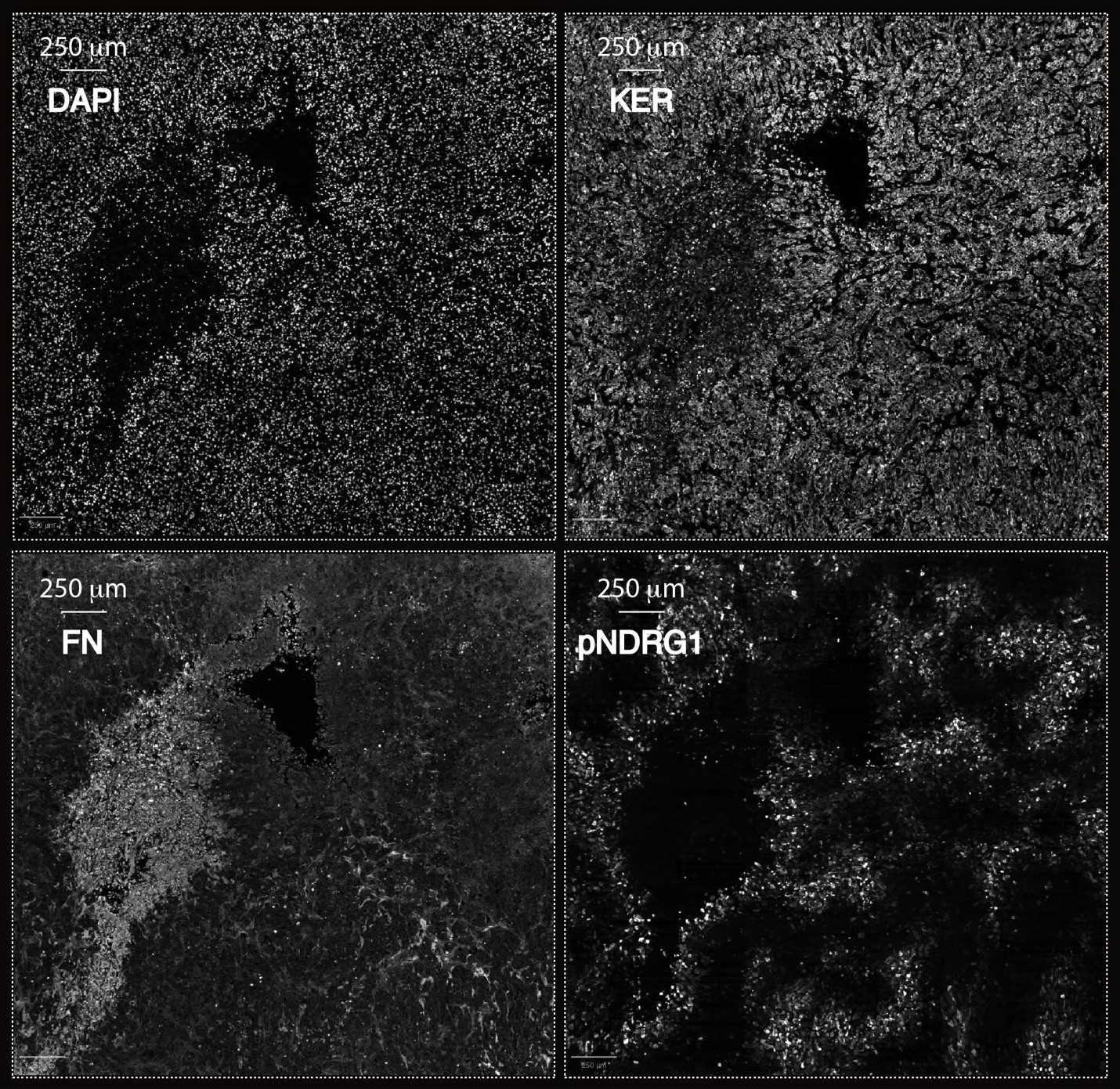
**

**Figure S1:** Representative whole-slide images from pancreatic ductal adenocarcinoma xenografts in grayscale, pNDRG1 dataset.

KER: Pan Cytokeratin, FN: Fibronectin, pNDRG1: phosphorylated N-Myc Downregulated Gene .


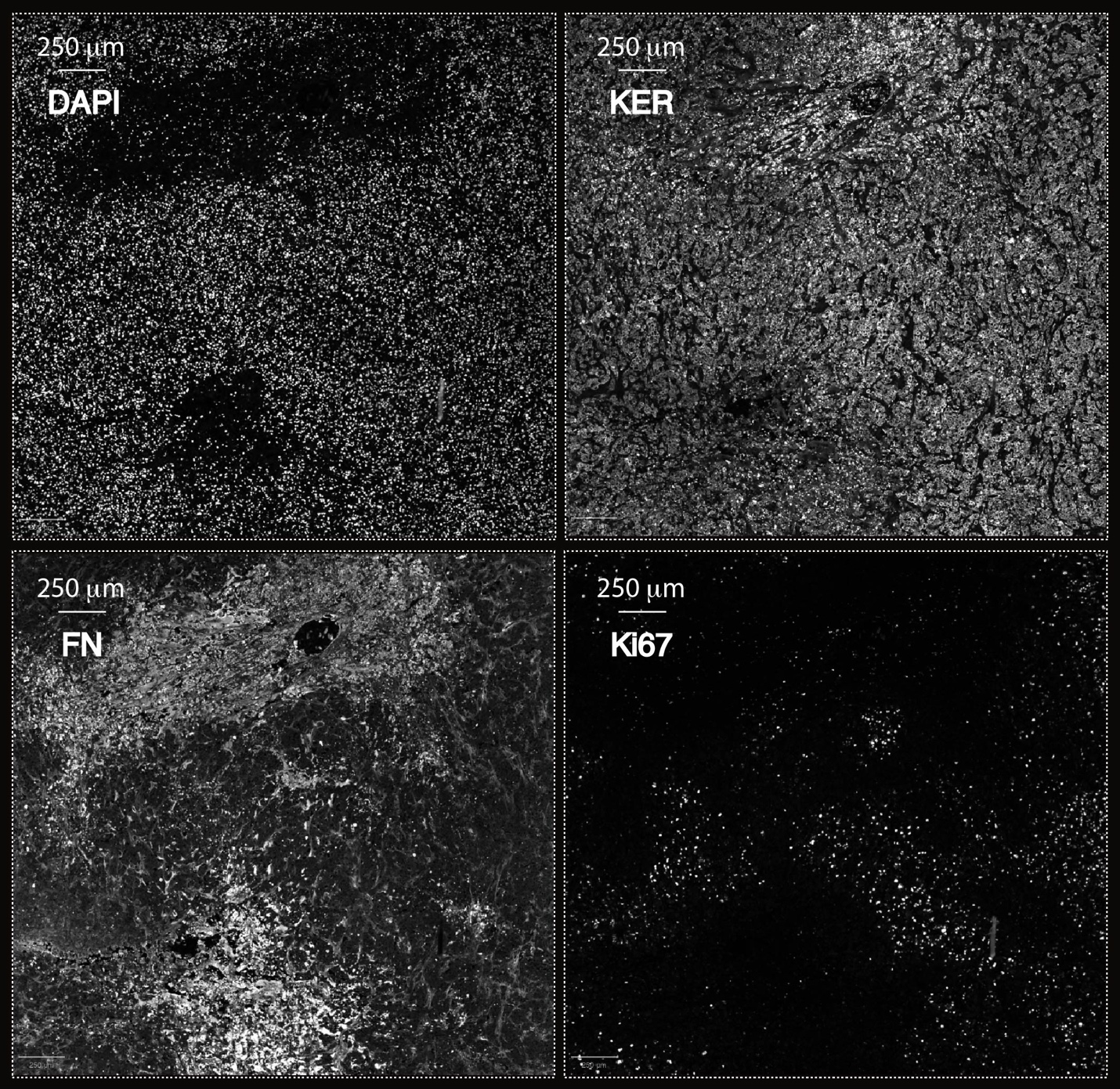


**Figure S2:** Representative whole-slide images from pancreatic ductal adenocarcinoma xenografts in grayscale, Ki67 dataset.

KER: Pan Cytokeratin, FN: Fibronectin.


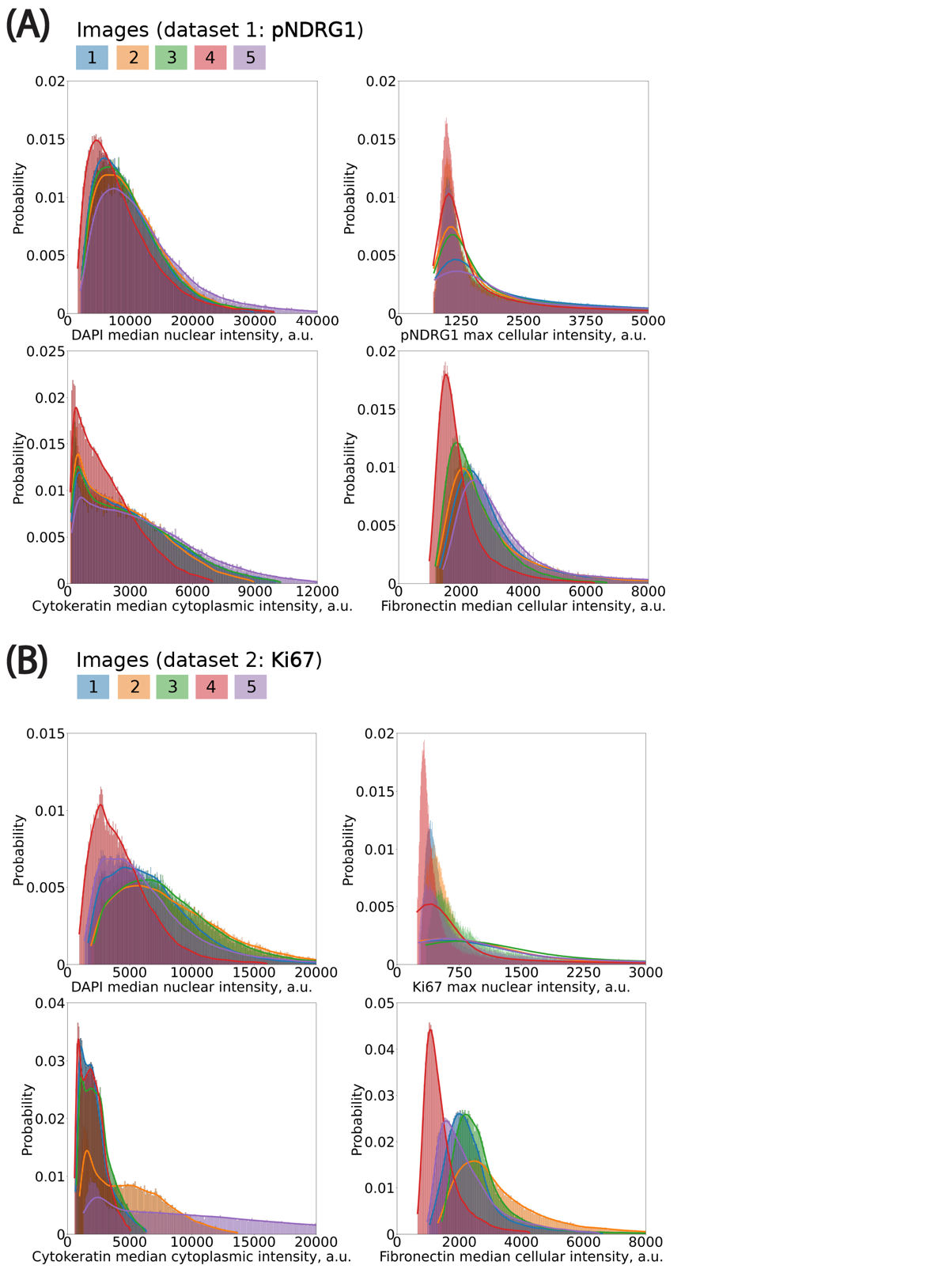


**Figure S3:** Histograms and kernel density estimations of cell measurements of interest.

**(A)** pNDRG1 dataset contains 5 images. Bin size (in µm): DAPI, 150; pNDRG1, 5; KER, 50; FN, 20. **(B)** Ki67 dataset contains 5 images. Bin size (in µm): DAPI, 50; Ki67, 5; KER, 75; FN, 40.

y-axis represents the percentage of total observations that fall into each bin.

***
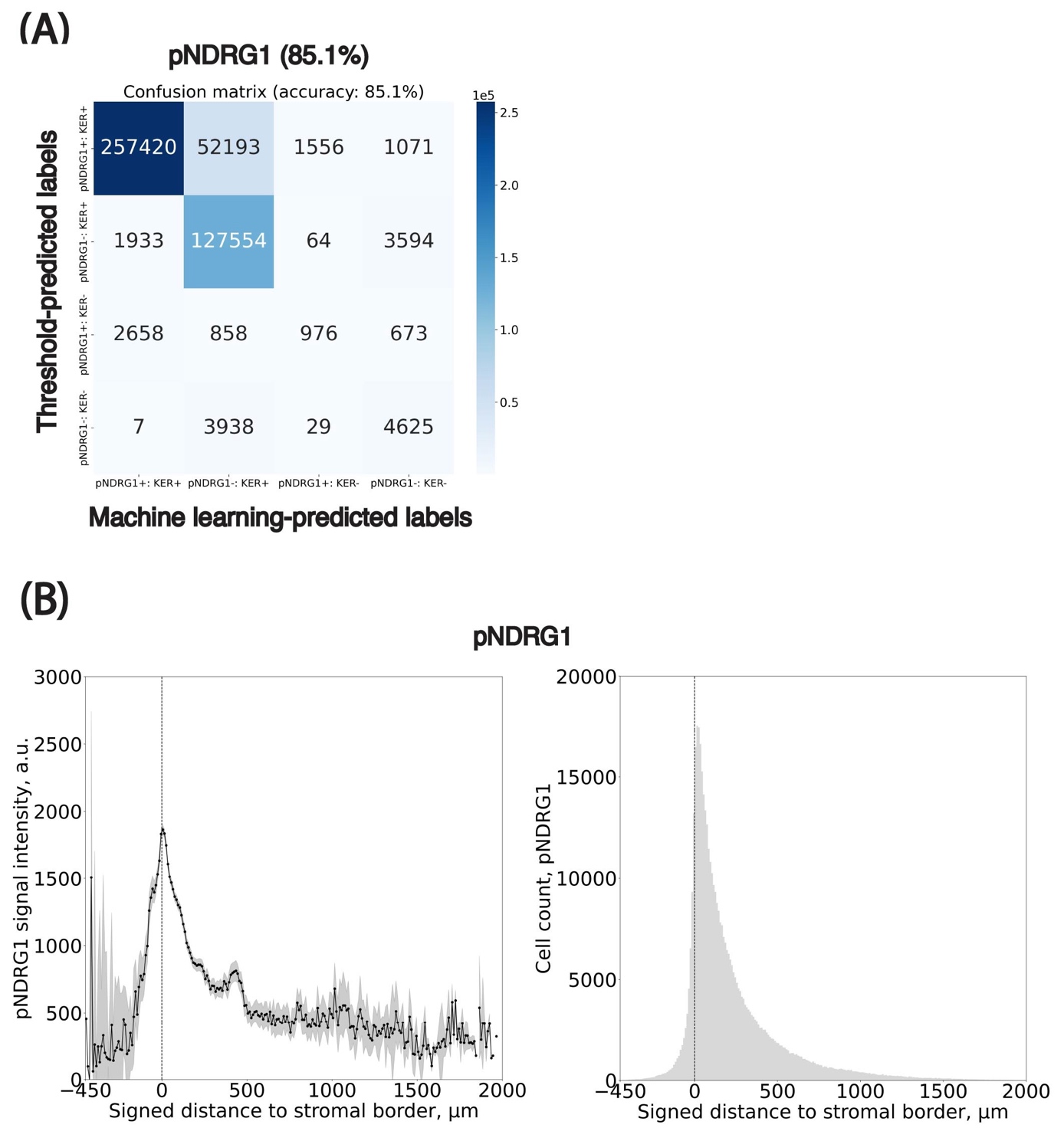
***

**Figure S4:** Full spatial distribution of pNDRG1-positive cancer cells relative to the stromal border.

**(A)** Comparison of machine learning-based and threshold-based classifiers across all possible class assignments from the following four classes: KER+, KER-, pNDRG1+ and pNDRG1-. **(B)** (left) Full spatial distribution of pNDRG1-positive cancer cells relative to the stromal border. Bin size: 10 µm. x axis is unclipped. Black dots represent the median value within a bin. Grey overlays indicate Standard Error of the Mean [SEM]. (right) Number of cells per bin. Same bin size.


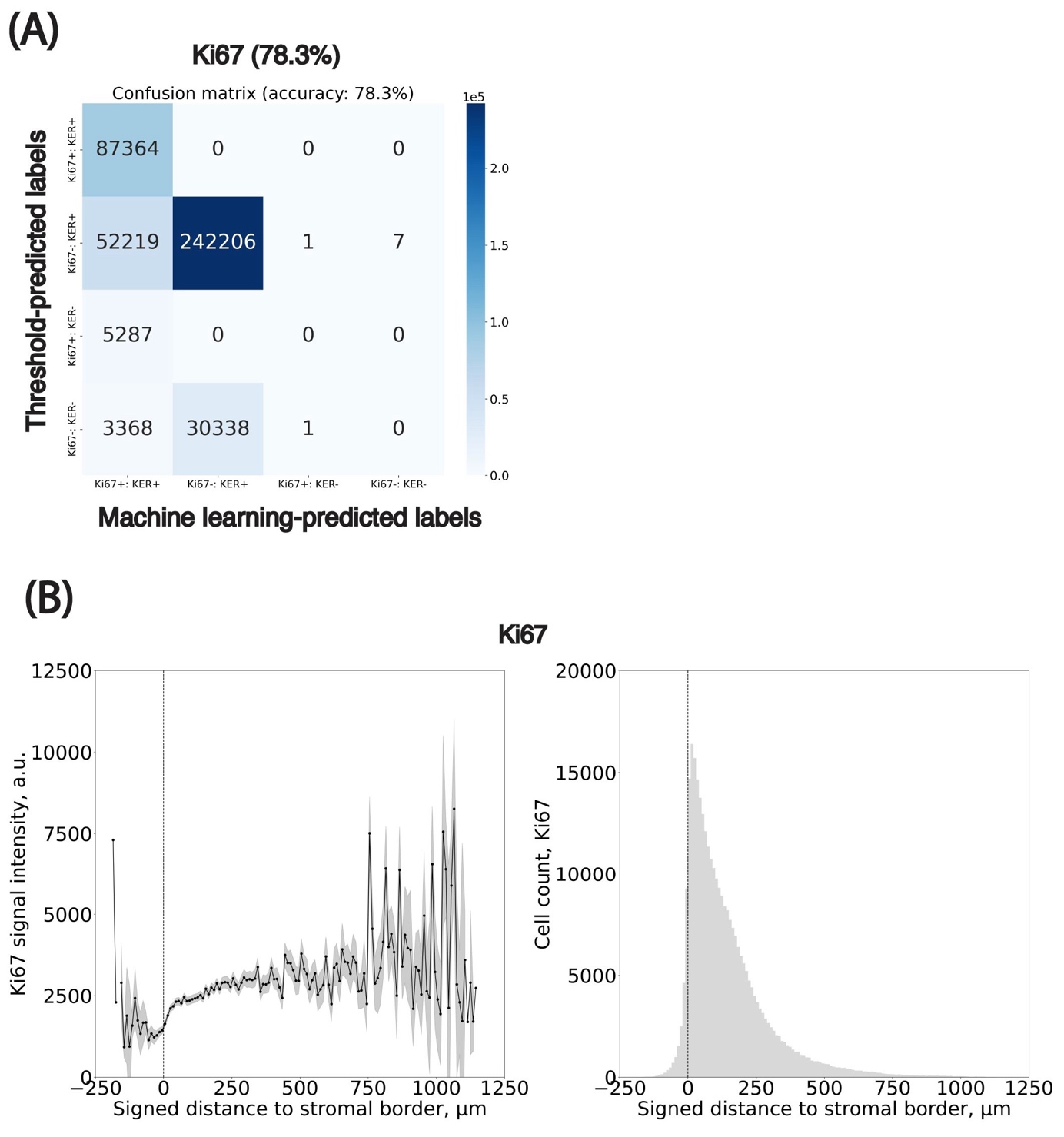


**Figure S5:** Full spatial distribution of Ki67-positive cancer cells relative to the stromal border.

**(A)** Comparison of machine learning-based and threshold-based classifiers across all possible class assignments from the following four classes: KER+, KER-, Ki67+ and Ki67-. **(B)** (left) Full spatial distribution of Ki67-positive cancer cells relative to the stromal border. Bin size: 10 µm. x axis is unclipped. Black dots represent the median value within a bin. Grey overlays indicate Standard Error of the Mean [SEM]. (right) Number of cells per bin. Same bin size.
